# When the blind curve is finite: dimension estimation and model inference based on empirical waveforms

**DOI:** 10.3389/fphys.2013.00075

**Published:** 2013-04-08

**Authors:** Fred Hasselman

**Affiliations:** Learning and Plasticity, Behavioural Science Institute, Radboud University NijmegenNijmegen, Netherlands

## A plea for strong inference

The recent research topic “*Fractal Analyses: Statistical and Methodological Innovations and Best Practices*” reveals there is no consensus among experts about the best procedure to estimate self-affine structure in trial and time series data. One of the recurring issues pertains to the validity of inferences based on analysis results about the physical change processes that generated the empirical waveforms. In this paper I argue that none of these approaches can be used to validate such inferences outside of the context of theory evaluation by strong inference (e.g., Platt, 1964). Two arguments warrant this claim: (1) All procedures make an assumption about the physics of the system under scrutiny. This is arguably most prominent in ARFIMA modeling, but associating an estimated scaling exponent to a fractal dimension is also based on assumptions (e.g., fGn vs. fBm; Mandelbrot and Van Ness, 1968); (2) given infinitesimal measurement resolution and infinite observation time, properties like dimension and self-affinity are not unique descriptors of a process, pattern or object (cf. Vicsek, 2001). Multiple mathematical models of physical processes can be constructed to generate a waveform with exactly the dynamical and invariant properties as observed in the finite sample (e.g., Mandelbrot, 2001; Kantz and Schreiber, 2003; Thornton and Gilden, 2005; Morrison, 2008).

The second issue pertains to a general problem of model-based inference: a good fit to a finite sample of measurement outcomes can never be conclusive in the evaluation of predictions by theories (cf. Roberts and Pashler, 2000, 2002; Fiedler et al., 2012). Using results of (fractal) analyses to answer questions about the physics of the observed system is an attempt to evaluate the ontology of a theory, ex post facto; let's leave ontology evaluation to the metaphysicians (cf. Poincaré, 1905, p. 211). The scientific method is not a competition for *mathematical models* constructed to produce the best fit to measurement outcomes; instead, *theoretical predictions* about the observed system compete for highest empirical precision and accuracy in order to gain scientific credibility.

In what follows I evaluate to what extent fractal analyses are used in the context of strong inference given the current empirical record of human physiology and performance. Subsequently I will explore what may be gained when implicit ontology falsification is removed from fractal analyses by introducing the concepts of intuitive dimension and informed dimension estimates.

## On fractal scaling and planetary orbits

Why should an accurate prediction by a theory be preferred over a good retrospective model fit? Models proposed to explain the orbit of Mercury (which displays a perihelion advance) present an interesting historical analogy. The orbit was accurately modeled by the classical geocentric models based on Ptolemy's Almagest (used from around 100–3500 CE; Toomer, 1984). These models assumed celestial objects moved around the earth on a celestial sphere that could host one or more local orbits or epicycles. The number of nested epicycles was simply varied until the predicted trajectory was sufficiently in accordance with the empirical record. Curiously, to the heliocentric models replacing Ptolemaic astronomy like Newton's theory of celestial mechanics, Mercury's orbit was an anomaly! No wonder that Einstein considered the accurate prediction of this anomaly the most important empirical test of his theory of general relativity (Einstein, 1916; Will, 2005).

This brief history of orbit modeling reveals that the theoretical perspective used to observe the empirical record changes one and the same reliably measured pattern from a good model fit, into an anomalous phenomenon into a critical benchmark for theory evaluation. Ptolemy's solution of adding epicycles to reconstruct the shape of a trajectory is essentially the same as adding weighted autoregressive, moving average, seasonally changing, or (fractionally) integrating components in a time series model. Those components are constructed into the model in order to create a better fit with a pattern in the data. This is allowed by mathematics, but their presence is not predicted by a theory of principles about physical change processes in living systems and this renders its scientific evaluation invulnerable to the presence of anomalies. Compare to Newton's closed theory of principles: “*In Ptolemy's case, if the orbit didn't fit, he could add other epicycles. But if an experiment does not fit in Newtonian physics, you don't know what you mean by the words*.” (Heisenberg interviewed by Kuhn, 1963, p. 24, February 27th).

In order to advance scientific knowledge about scaling phenomena in living systems a program of strong inference that aims to produce closed theories of principles is needed. In order to reach this goal, empirical inquiries need to go beyond describing scaling phenomena in different populations in the context of impaired performance or pathology (e.g., Goldberger et al., 2002; Gilden and Hancock, 2007; West, 2010; Wijnants et al., 2012a). Several recent studies reveal scaling phenomena can be brought under experimental control, which is essential for a program of strong inference (e.g., Kello et al., 2007; Wijnants et al., 2009; Van Orden et al., 2010; Correll, 2011; Holden et al., 2011; Kuznetsov et al., 2011; Stephen et al., 2012). The diverging theoretical predictions examined in most studies reveal that the observed waveforms are more likely to originate from interaction-dominant complexity than from component-dominant mechanics (also see Turvey, 2007; Kello et al., 2010; Diniz et al., 2011).

A closed theory should account for most phenomena in the existing empirical record. A first step was recently made in which it was shown that the well-known speed-accuracy tradeoff in human performance is meaningfully related to the emergence of self-affine structure via nested timescales (Wijnants et al., 2012b). At the current level of scientific understanding it seems reasonable to ask of those who insist models based on AR-processes provide a parsimonious explanation of fractal scaling (e.g., Wagenmakers et al., 2005; Torre and Wagenmakers, 2009; Stadnitski, 2012), to provide experimental evidence that can validate their claims.

As stated above however, most claims about scaling phenomena based on fractal analyses are prone to implicit ontology falsification. In what follows, I will suggest an approach to dimension estimation that is based on intuitions about the geometry of a curve rather than on known mathematical models of change processes. I will focus on the mono-fractal case and show that a consistent conversion scheme for common estimates of self-affine structure is possible when using this notion of dimension.

## Finite self-affinity: the blind curve and the perimeter walk

Dimension is an intrinsic property of a mathematical object that indicates to what extent it occupies the topological space in which it is embedded. A dimension estimate that is based on the properties of an empirical waveform can be defined as a finite walk in the plane that never forms a perimeter. Formally, this is a self-avoiding open curve dividing a bounded plane in two unconnected regions (i.e., it is not a Jordan Curve). Note that the properties of the curve have a physical origin: it is self-avoiding and open due to the arrow of time and because observation duration and measurement outcomes are finite, the planar topology is bounded.

**Figure 1:**
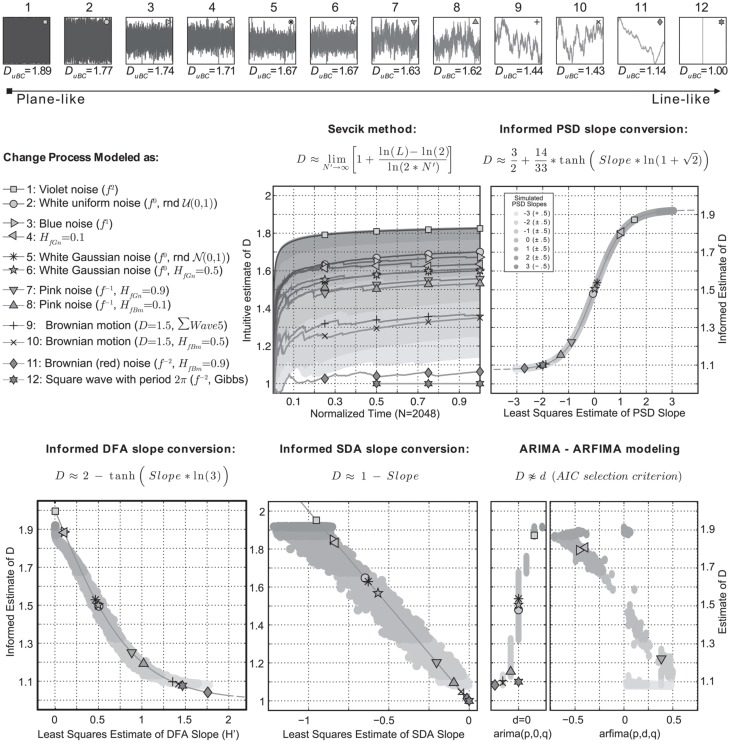
**The top row shows 12 waveforms of 2^11^ data points embedded in a unit square and ordered according to their planar extent estimated by *D*_*uBC*_.** This is a 2D box-counting dimension estimate calculated from a binary image (2×N by 2×N logical matrix) of the graph of the waveform. The same relative order was recovered using Sevcik's method, which estimates *D* from the waveform based on curve length *L* and data length *N* − 1 (*N′*). The gray-scale coded areas refer to Sevcik's estimate for 12100 simulated series with PSD slopes varying from −3 to 3. In the other graphs, the gray areas show the estimate of *D* based on PSD slope. The conversion formulas relate self-affinity exponents to *D* informed by known values of these exponents for power laws in spectral density. No conversion could be found for ARFIMA modeling. See text for details.

Estimation procedures derived from formal definitions of dimension respect an intuitive geometric notion of a scaling of bulk with size (Theiler, 1990). Using the definition above, the intuitive concept to quantify would be a characterization of the waveform as line-like or plane-like, hence *planar extent* (e.g., Higuchi, 1988; Katz, 1988; Raghavendra and Dutt, 2010). Sevcik (1998) introduced such a dimension estimate based on the Hausdorff-Besicovitch dimension (Hausdorff, 1919; Xiao, 2008). It involves a double linear transformation of the axes embedding the waveform in a unit square of size N by N. Its length can be calculated as the sum of the Euclidean distance between points on the normalized curve. The graph entitled “Sevcik method” in Figure 1 shows the equation used to approximate *D* based on number of observation intervals (N–1) and curve length *L* (for details see Sevcik, 1998, 2006). Across the top of Figure 1, twelve different waveforms are shown that were analyzed for self-affine structure (see caption for details). The waveforms were generated using freely available Matlab scripts^1^.

## Intuitive dimension

The dimension estimates for the 12 waveforms based on Sevcik's mehod are numerically different from *D*_*uBC*_, as well as from known exact values, the goal however is to achieve relative consistency. Processes that generated waveform 9 and 10 have known *D* = 1.5, which is equal to a sequence of random numbers drawn from a Normal distribution (i.e., waveform 5, waveform 10 is its cumulative sum). Using Sevcick's method however, both waveforms are classified as a Brownian noise. If this waveform were known to be physiological and medical in nature, the constrained dynamics associated with Brownian noise would lead to profoundly different conclusions about the health and well-being of the patient in question compared to blindly interpreting the limit values *D* = 1.5 and *H* = 0.5 (e.g., Goldberger et al., 2002; Van Orden et al., 2009). The gray-scale areas represent Sevcik's estimate for 12100 simulated series with ideal spectral slopes ranging from −3 to 3. Note that at 25% of the data length (first set of markers) the relative ordering according to *D_uBC_* is recovered for almost all waveforms.

## Informed dimension estimates

The other graphs represent self-affinity exponents estimated using the power spectrum (PSD), detrended fluctuation analysis (DFA), standardized dispersion analysis (SDA), and ARFIMA (modeling strategy: Reisen and Lopes, 1999; Silva et al., 2006). The informed estimates of *D* refer to conversions of the self-affinity indices obtained for the 12 waveforms. ARFIMA modeling did not provide a consistent conversion scheme therefore the differencing parameter was plotted against the PSD based estimates. The gray-scale coded regions in these plots refer to the PSD slope estimates for the 12100 simulated series. These areas thus display the relation between the DFA, SDA, and ARFIMA self-affinity indices and the PSD slope based estimate.

The equations suggested for PSD and DFA indices produce approximations of well-known (*H*, *D*) pairs and should not be confused with an analytic solution. For SDA the known formula *1-Slope* yields relatively consistent results, a problem is that for some ranges of PSD slopes, the SDA indices are the same. ARFIMA modeling by the AIC selection criterion preferred models without self-affine structure (*d* = 0) of varying order (*p* = 0–2, *q* = 0–2) for the majority of simulated PSD slope series (ARIMA 42.8% vs. ARFIMA 20.6%). The remaining series produced fit errors (see Supplementary Materials for details).

Most waveforms in Figure 1 get assigned a value for *D* that is in accordance with their planar extent as indicated by *D*_*uBC*_. Waveform 11 is more line-like than waveform 9 and 10, which both map closely to known *D* of Brownian noise. Expected exceptions are waveforms 2 and 12. A sequence of random numbers drawn from a uniform distribution (waveform 2) has a PSD slope of zero. Taking the Fourier transform of a square wave (waveform 12) gives a frequency spectrum of odd harmonics only, with a slope of exactly −2 (the Gibbs phenomenon). Another expected result is that ARFIMA is preferred for series produced as H_fGn_ (except *H*_fGn_ = 0.5).

## To self-affinity … and beyond!

It seems possible to remove implicit assumptions about system ontology from fractal analysis by defining dimension as the planar extent of a finite curve. Direct estimates based on curve length and 2D box-counting provide a consistent relative ordering on this dimension. An informed conversion scheme using estimates of self-affine structure obtained from PSD, DFA, and SDA analyses give similar results. Some exceptions were predicted, but ARFIMA modeling could not be included in the approach due to inconsistent analysis results. A mono-fractal perspective was explored here, but there is no reason to assume it cannot be extended to the multi-fractal framework as well.

Exact numerical similarity of estimates is sacrificed for the convergence of estimates to a similar relative ordering. This sacrifice is acceptable given that in principle, even the best estimates of dimension and self-affinity leave us blind to the physical processes that generated the waveform. I suggest that claims about the physics of the system need to be evaluated by comparing the empirical accuracy of theoretical predictions in a program of strong inference, not by comparing fit indices.

## Footnotes

^1^The scripts are available at http://fredhasselman.com in the section Supplementary Materials.
